# Mr. Henry Collen Ensor

**Published:** 1910-09-17

**Authors:** 


Mr. Henry Collen Ensor, of Cardiff, has died at the age of fifty-one. He studied at Guy's Hospital, and qualified as a member of the Royal College of Surgeons, England, and as an L.S.A. in 1885. Mr. Ensor had held appointments at the Cardiff Eye and Ear Hospital and the Newport Hospital, and was ophthalmic surgeon to the Cardiff Infirmary, the Cardiff Deaf and Dumb Institution, and the Royal *Hamadryad* Seamen's Hospital at Cardiff.

